# The mode and dynamics of glioblastoma cell invasion into a decellularized tissue-derived extracellular matrix-based three-dimensional tumor model

**DOI:** 10.1038/s41598-018-22681-3

**Published:** 2018-03-15

**Authors:** IlKyoo Koh, Junghwa Cha, Junseong Park, Junjeong Choi, Seok-Gu Kang, Pilnam Kim

**Affiliations:** 1 Department of Bio and Brain Engineering, KAIST, Daejeon, 34141 Korea; 2 Department of Neurosurgery, Brain Tumor Center, Severance Hospital, Yonsei University College of Medicine, Seoul, 03722 Korea; 3 Department of Pharmacy, College of Pharmacy, Yonsei Institute of Pharmaceutical Sciences, Yonsei University, Incheon, Republic of Korea

## Abstract

Glioblastoma multiforme (GBM) is the most common brain tumor with very aggressive and infiltrative. Extracellular matrix (ECM) plays pivotal roles in the infiltrative characteristics of GBM. To understand the invasive characteristic of GBM, it is necessary to study cell-ECM interaction in the physiologically relevant biomimetic model that recapitulates the GBM-specific ECM microenvironment. Here, we propose biomimetic GBM-specific ECM microenvironment for studying mode and dynamics of glioblastoma cell invasion. Using tissue decellularization process, we constructed a patient tissue-derived ECM (pdECM)-based three-dimensional *in vitro* model. In our model, GBM cells exhibited heterogeneous morphology and altered the invasion routes in a microenvironment-adaptive manner. We further elucidate the effects of inhibition of ECM remodeling-related enzymatic activity (Matrix metalloproteinase (MMP) 2/9, hyaluronan synthase (HAS)) on GBM cell invasion. Interestingly, after blocking both enzyme activity, GBM cells underwent morphological transition and switch the invasion mode. Such adaptability could render cell invasion resistant to anti-cancer target therapy. There results provide insight of how organ-specific matrix differentially regulates cancer cell phenotype, and have significant implications for the design of matrix with appropriate physiologically relevant properties for *in vitro* tumor model.

## Introduction

Invasion and dissemination of cancer cells cause migration of neoplastic cells into surrounding tissues, resulting in mortality in tumor patients^1–3^. In particular, cancer cell invasion is of enormous clinical importance, since it involves both distant metastasis and local spreading, whereby cancer cells degrade and migrate through the tissue. The significance of cancer cell invasion is evident in the glioblastoma multiforme (GBM), which shows infiltrative and rapid growth into the surrounding brain tissue. The invasive and migratory characteristics of GBM cells provide a wealth of information about tumors within a patient^3^.

Three-dimensional (3D) *in vitro* culture systems are increasingly employed to assess cell-ECM interactions and invasion of tumor cells. Indeed, cell behavior and responsiveness are dramatically different in a 3D physiological environment versus two-dimensional (2D) Petri dish conditions^4,5^. Importantly, in 3D cultures, ECM is an essential determinant of the cellular response to invasion and migration processes^6^. Brain ECM has a distinct composition from ECM in other tissues and organs, with a low stiffness and loosely connected cellular network. The ECM component of brain tissues contains high amounts of hyaluronic acid (HA), glycosaminoglycans (GAGs), and proteoglycans, but lacks fibrous materials such as collagen, fibronectin, etc^7,8^.

In fact, the interaction between GBM cells and unique extracellular environment of the brain could impact on the invasive characteristics of GBM cells. Accumulated experimental and clinical data demonstrate that invasion of GBM cells is regulated by several environmental mechanisms that facilitate the spread of these tumors^9,10^. For example, the invasion pattern of malignant GBM is associated with distinct anatomic pathways following myelinated fiber tracts and blood vessels^4,11^. In addition to anatomical and physical aspects, there is accumulating evidence that specific extracellular matrix (ECM) components (such as hyaluronan, vitronectin, and tenascin-C) are unregulated at the border of the spreading GBM, and this may alter cellular invasiveness^7,10,11^. Because molecular guidance cues during cell invasion are often dependent on the ECM, the underlying mechanism of glioblastoma invasion and the GBM-specific ECM microenvironment represent potential targets for treating GBM.

Despite the significance of a brain-specific ECM microenvironment for understanding GBM cell invasion, most culture systems have so far utilized matrix materials (either synthetic or derived from animals)^12–15^. Such systems have limited utility for expanding the use of *in vitro* models, to enhance the efficiency of anti-cancer drug screening or understand the mechanisms of invasion.

To address these issues, we develop a patient tissue-derived decellularized ECM (pdECM) based 3D tumor model as a physiologically relevant *in vitro* system. To show that a patient-derived ECM has impacts on cancer cell behaviors, we assessed invasion characteristics of GBM cells within decellularized ECM compared to collagen matrix *in vitro*, with a specifically focus on morphology and dynamics of invasion. The disseminated GBM cells exhibited heterogeneous morphology and invasion mode within ECM but were drastically different within the collagen matrix. By blocking ECM remodeling-related enzymatic activities, GBM cells facilitated change in the disseminated cell and underwent the alteration of invasion strategy. Our results highlight the potential of patient-derived tissue-specific ECM for use in *in vitro* tumor model.

## Results

### Characterization of Decellularized Patient-derived Extracellular Matrix (pdECM)

**Figure 1:**
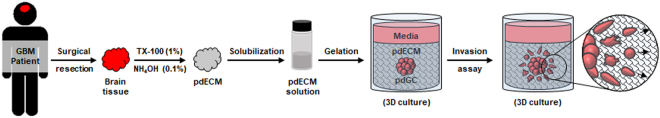
Schematic illustration of the patient tissue-derived extracellular matrix (pdECM)-based three-dimensional (3D) tumor model.

**Figure 2:**
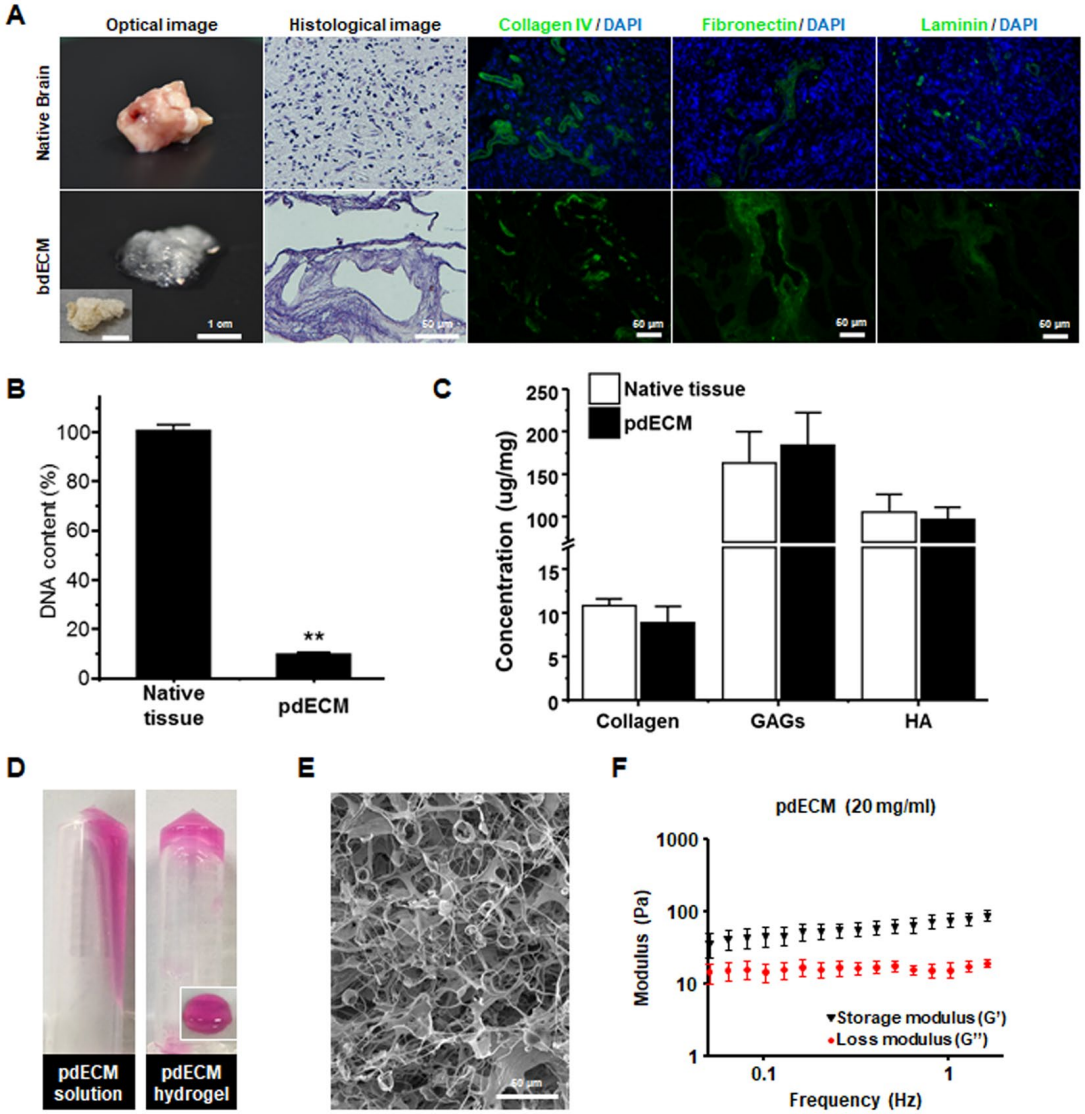
Characterization of patient tissue-derived extracellular matrix (pdECM). (**A**) Optical and histological images of native brain tissue and pdECM. (**B**) Quantification of DNA content in native brain tissue and pdECM (**p < 0.01). (**C**) Quantification of ECM components (collagen, glycosaminoglycans [GAGs]) in native brain tissue and pdECM (n = 4), (*P < 0.05; **p < 0.01). (**D**) Gelation of pdECM pre-gels solution. (**E**) Scanning electron microscopy image (SEM) of pdECM hydrogel. (scale bar = 100 *μ*m). (**F**) Mechanical property of pdECM hydrogel.

To investigate cell-ECM interaction and the invasion phenotype of glioblastoma cells, we developed a physiologically relevant *in vitro* 3D tumor models that reconstruct the GBM-specific ECM microenvironment (Fig. 1). We utilized decellularized patient tissue-derived ECM (pdECM) as a matrix material that preserves the complex mixture of *in vivo* ECM components and structures. The pdECM was obtained from GBM patients’ brain tissues (n = 15) via decellularizing solution (1% Triton X-100, 0.1% NH_4_OH). Histological analysis showed that the nucleus and/or cellular components were removed completely from native tissue. Immunofluorescence analysis indicated that ECM components, including collagen IV, fibronectin and laminin, were preserved after decellularization (Fig. 2A). DNA content analysis showed that the amount of DNA in pdECM was 8.9% of that in native tissues (p < 0.01) (Fig. 2B). The contents of ECM components, including collagen, GAGs, and HA were not significantly changed after decellularization (Fig. 2C). After decellularization, pdECM was solubilized by pepsin digestion to reconstruct a 3D hydrogel. To promote polymerization of pdECM, we added collagen pre-gel solution (4 mg/ml) to a final pdECM solution (20 mg/ml). In that, pdECM-based scaffolds contained 10% collagen of their original volume (10: 1, pdECM/collagen; v/v). The pdECM pre-gel solution (pdECM pre-gel) was acidic (pH 2~3) before encapsulating the pdGCs. When the pdECM pre-gel was adjusted to physiological conditions (pH 7), it behaved like a thermo-sensitive hydrogel. The pH-adjusted pdECM pre-gel was incubated in 37 °C for 0.5 to 1 h, transforming the pdECM pre-gel to hydrogels (Fig. 2D). Scanning electron microscopy (SEM) images showed the microscopic structure of the pdECM-based hydrogel. The matrix had a porous and fibrous structure composed of nanofibers forming larger microscale fibrils (Fig. 2E). The elastic modulus of each sample was measured under oscillation conditions after forming a hydrogel. The average modulus of the pdECM hydrogel is 78.09 ± 29.22 Pa (Fig. 2F) that was comparable to that of brain tissue^16^. We, therefore, conclude that our decellularized pdECM is acceptable as a human brain-ECM for *in vitro* 3D tumor model.

### Invasion of pdGCs in pdECM-based three-dimensional (3D) tumor model

**Figure 3:**
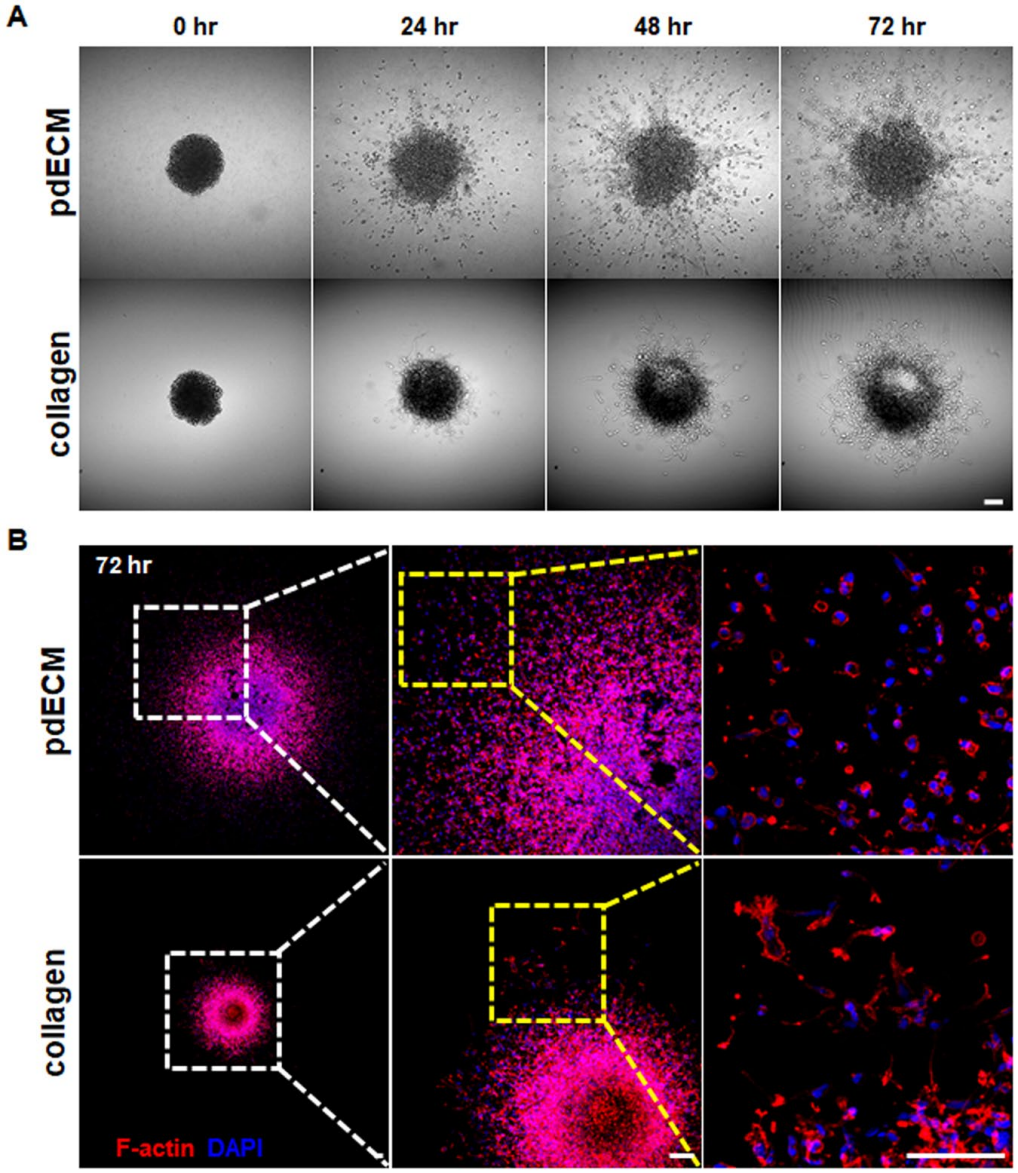
Time-laps invasion image of pdGCs in pdECM-based 3D tumor model. (**A**) Representative time-lapse images of invasion by pdGCs into pdECM and collagen matrices (scale bar = 100 *μ*m). (**B**) Filamentous actin (F-actin) staining of pdGCs in pdECM and collagen matrices.

**Figure 4:**
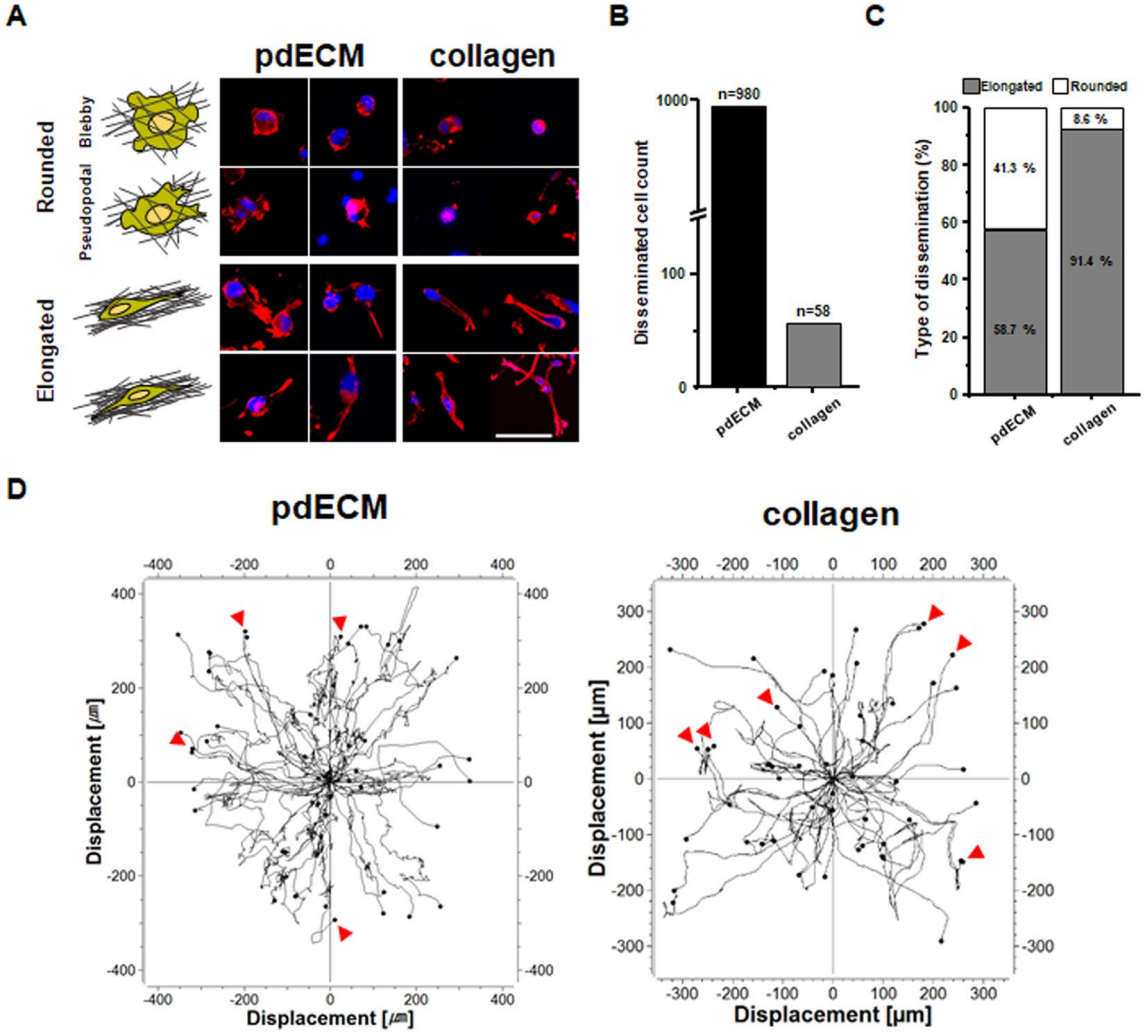
pdGCs display complex invasion phenotype in the pdECM-based 3D tumor model. (**A**) Representative images of cellular morphologies in each hydrogel (scale bar = 100 *μ*m). (**B**) Quantitative analysis of disseminated cells count. (**C**) Quantitative analysis of dissemination distribution by morphological type. (**D**) Cell tracking analysis of pdGCs in each hydrogel.

We next explanted patient-derived GBM cells (pdGCs) into pdECM and developed *in vitro* 3D tumor models. pdGCs were prepared according to previous literature on cell isolation from GBM patient tissues^17^. Using this model, we explored how the ECM microenvironment impacts on the kinetics of cancer cell invasion and dissemination. Figure 3A displays representative time-lapse images of the invasive patterns of pdGCs in pdECM. The cells showed high invasiveness into the adjacent matrix, and the core of the tumorsphere had spread widely after 3 days. In contrast, pdGCs showed relatively limited migration in collagen hydrogel compared to pdECM (Fig. 3). In both pdECM and collagen matrix-based tumor models, we observed disseminating cells at the protrusive border that exhibited single-cell migration behavior, characterized by a lack of cell-cell interactions. The invading cells showed significantly different morphologies depending on the culture matrices. The disseminated cells exhibited heterogeneous morphologies, including rounded and elongated shapes (Figs 3 and 4, Movie S1).

To investigate this further, we stained a filamentous actin (F-actin) structure of the invaded cell with fluorescent phalloidin (red) (Fig. 3B). We classified and counted these morphologies according to the cell body to protrusion ratio (less than 1:1, rounded morphology; other, elongated morphology [data not shown]). F-actin rich cellular protrusions (i.e., invadopodia) were seen in pdGCs cultured in collagen matrix rather than pdECM. In pdECM, the total number of disseminated cells was approximately 17-fold higher compared to that in collagen (Fig. 4B). Moreover, pdGCs cultured in pdECM exhibited both elongated (58.7%) and rounded (41.3%) shapes. In contrast, pdGCs cultured in collagen matrix disseminated in a mesenchymal fashion and appeared as elongated cells (91.4%) (Fig. 4C). Thus, the pdGCs cultured within pdECM showed significantly heterogeneous morphologies (elongated or rounded shape), single-cell migration behavior and indistinct cellular margins. This result is consistent with previous research showing that GBM cells have high morphological heterogeneity^18^.

**Figure 5:**
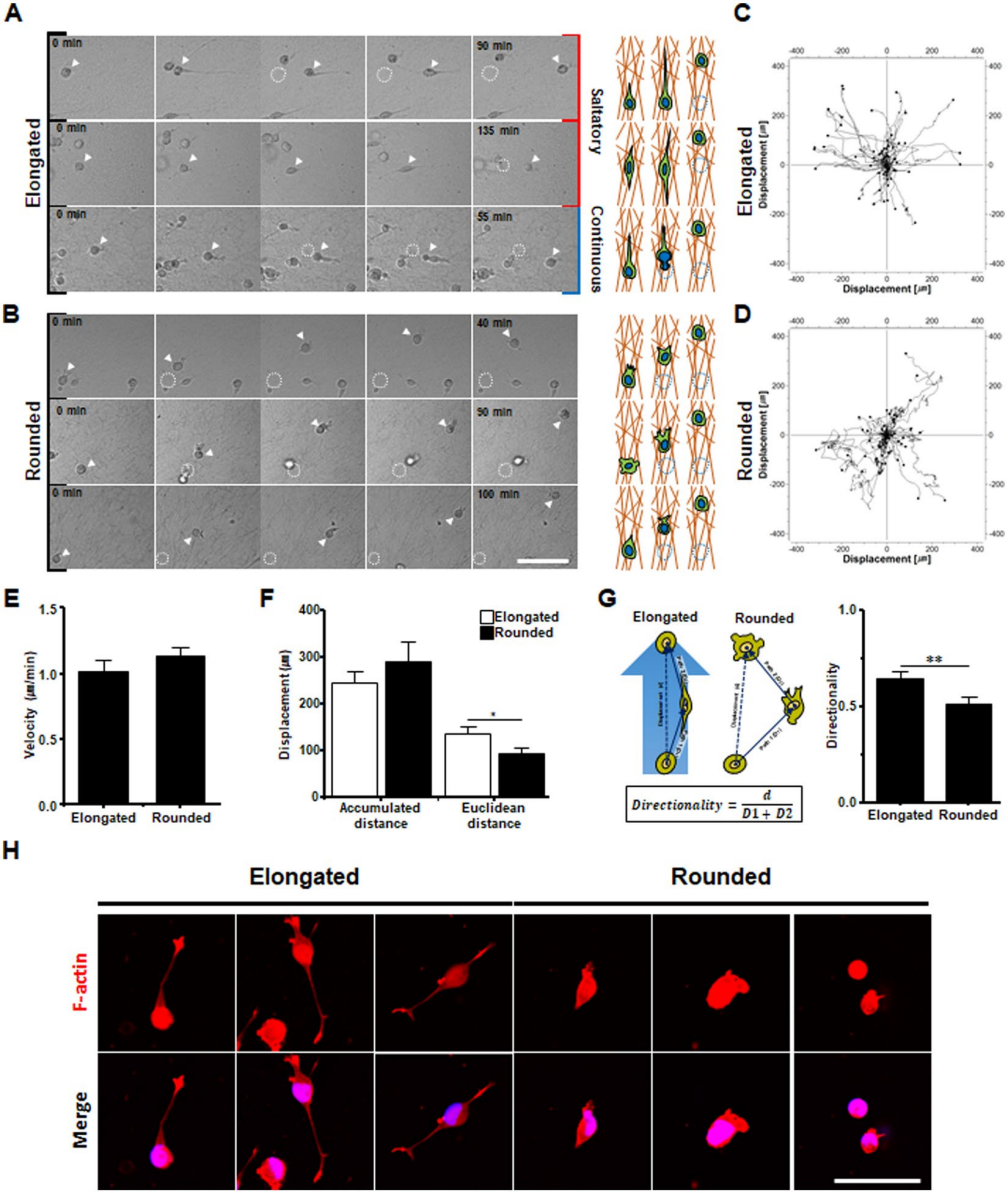
Two distinct invasion strategies in a patient-derived ECM-based 3D culture model. (**A**,**B**) Representative single-cell trajectory images of pdGCs in pdECM hydrogels (scale bar = 100 *μ*m). (**C**,**D**) Cell tracking analysis of pdGCs by cellular morphology in pdECM hydrogel. (**E**) Velocity profiles of pdGCs by cellular morphology in pdECM hydrogels. (**F**) Difference between accumulated and Euclidean distances by cellular morphology in pdECM hydrogels (*p < 0.05). (**G**) Directionality of invading pdGCs by cellular morphology in pdECM hydrogels (**p < 0.01). (**H**) Representative images of pdGC distribution (F-actin) in pdECM hydrogels (scale bar = 50 *μ*m).

To further identify the ECM-dependent invasion dynamics of cells, we performed a cell-tracking analysis. In Fig. 4D, the pdGCs showed constant radial dissemination and/or migration into the surrounding matrix in a random manner in the pdECM hydrogel, as well as a relatively high migration velocity (0.97 ± 0.06 μm/min) (Movie S1d). In collagen matrix, GBM cells migrated and/or infiltrated into the adjacent matrix with a relatively slow migration velocity (0.33 ± 0.02 μm/min) and we occasionally observed cells that were slowing down or pausing temporarily during migration (Fig. 5A, Movie S1a,b). In some cases, the trajectories of invading pdGCs overlapped partially or completely between pdECM and collagen (Fig. 4D; red arrow), which presumably indicates the formation of invasion-tracks generated by front leading cells. The invasion-tracks were more frequent in cells cultured in collagen matrix versus those cultured in pdECM (Movie S1c,e). Our results imply that pdECM facilitates the dissemination of pdGCs into the surrounding matrix with high migration velocity (0.97 ± 0.06 μm/min) and in a random manner.

Next, we assessed whether cell migratory behavior in a single cell level might be different depending on cell morphology (Fig. 5, Movie S2). For the elongated morphology type, individual cell trajectories showed high directional persistence and the cells spread radially. During the invasion process, the elongated cells exhibited directional persistence, that is, sustained migration in the same direction (Fig. 5C). However, for the rounded morphology type, cells showed increased tortuosity and a decrease in persistent directional migration (Fig. 5D). To further elucidate the dependence of cell-shape on cell invasion behavior, we quantified the migration speed and directionality of each cell morphology type. Although there was no difference in the migration speed or total distance migrated (accumulated distance), the Euclidean distance (linear distance between the start and endpoint of a cell) showed significant differences between rounded and elongated cell morphology types (Fig. 5E, F). Directionality, which describes the orientation of cell migration (Euclidean distance divided by the accumulated distance), was significantly increased for the elongated cell morphology type (Fig. 5G). Elongated cells exhibit thin and long filopodia, allowing them to move in a directionally persistent manner. Herein, the elongated cells exhibited two types of migratory behavior. First, some cells showed discontinuous and saltatory migration, during which the cells interact with the pdECM and form thin and long leading protrusions (uni-/bi-directional) in the direction of movement. Once a cell forms a leading protrusion, it starts to migrate and shows complete adhesion to the surrounding ECM. Afterward, the protrusive edge shrinks rapidly and the cell relocates (Fig. 5A, top, middle, Movie S2a,b). In addition to saltatory migration, some cells displayed continuous migration (Fig. 5A, bottom, Movie S2c). Rounded cells displayed an irregular and rapidly changing morphology, forming poorly defined, short protrusions at the leading edge of the cell (Fig. 5A, right, Movie S2d,e,f). These results indicated that there is a correlation between the directional persistence of invading cells and the formation of protrusions during migration. Taken together, we identified cell-shape dependent migration behaviors. Elongated cells formed thin and long filopodia and showed invasion in a persistent direction, while rounded cells exhibited bleb-like structures and formed multiple, poorly defined protrusive edges.

### Gene expression of pdGCs in pdECM-based three-dimensional (3D) tumor model

**Figure 6:**
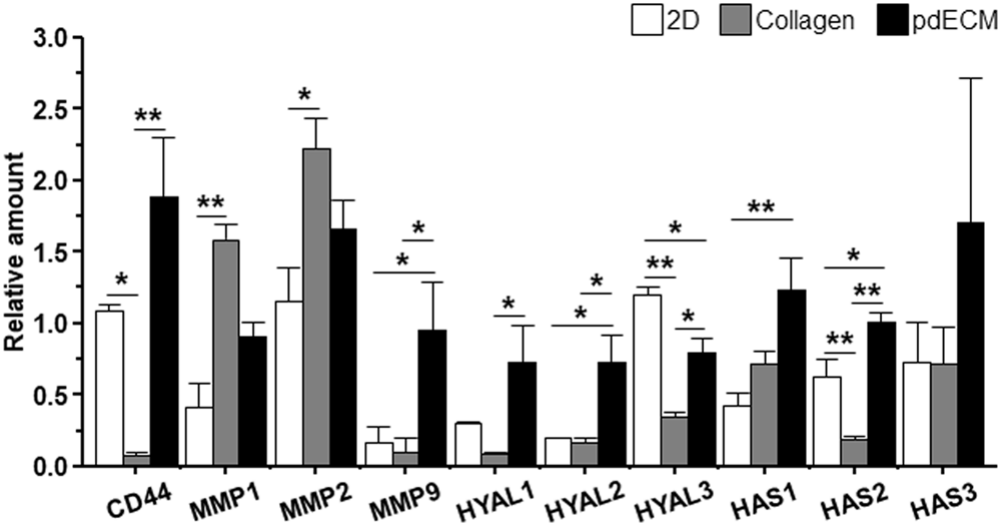
Quantitative polymerase chain reaction (qPCR) analysis of pdGCs in different ECM microenvironments. qPCR analysis of the expression levels of ECM-related genes in 2D, collagen and pdECM hydrogels (n = 3~5; Asterisks indicate a significant difference statistically by ANOVA (*p < 0.05; **p < 0.01)).

Brain ECM is composed of typical ECM proteins (ex., laminin, type-IV collagen, and fibronectin) and an HA-enriched matrix with associated proteoglycans and glycoproteins. Especially, in malignant GBM, HA contains higher-than-normal amounts of brain ECM^7,8^. Increased HA in GBM promotes the invasive and infiltrative phenotype by activating a number of coordinated cellular programs^7,10^. This raises the possibility that an HA-enriched environment in our pdECM-based 3D tumor model might play an important role in pdGCs invasion and facilitate complex invasion phenotypes. Moreover, during tumor progression, tumor cells apparently produce enzymes that destroy and remodel the matrix microenvironment, thus permitting invasion into surrounding tissues^2,3^. We, therefore, analyzed the encoded HA-related genes and matrix metalloproteinases (MMPs), including the HA-receptor (CD44), MMP 1, 2 and 9, hyaluronan synthases (HASs) 1, 2 and 3 and hyaluronidases 1, 2 and 3 (Hyal1/2/3) (Fig. 6). We collected mRNA from pdGCs cultured in 2D, collagen, and pdECM environments after 3 days. By quantitative real-time (qRT) polymerase chain reaction (PCR) analysis, we identified that MMP1, an enzyme that breaks down collagen type 1, was the most highly expressed cell culture within the collagen matrix. CD44, HAS, Hyal1/2 and MMP9 were significantly increased in pdGCs cultured within pdECM. As we mentioned previously, our pdECM contained various components of brain ECM (e.g., HA, GAGs, Col IV, etc.). Therefore, over-expressed HAS, Hyal1/2 and MMP9 (Col IV-cleaving MMP) genes might contribute to ECM remodeling during cell invasion. In particular, increased levels of HAS in pdECM resulted in newly synthesized HA that sequentially interacted with surface receptors of GBM cells. Therefore, the up-regulated activity of enzymes, such as HAS and MMP, might be correlated with increased invasion of GBM cells in pdECM.

### Effects of MMP9 and HAS2 Inhibitors in pdECM-based three-dimensional (3D) tumor model

**Figure 7:**
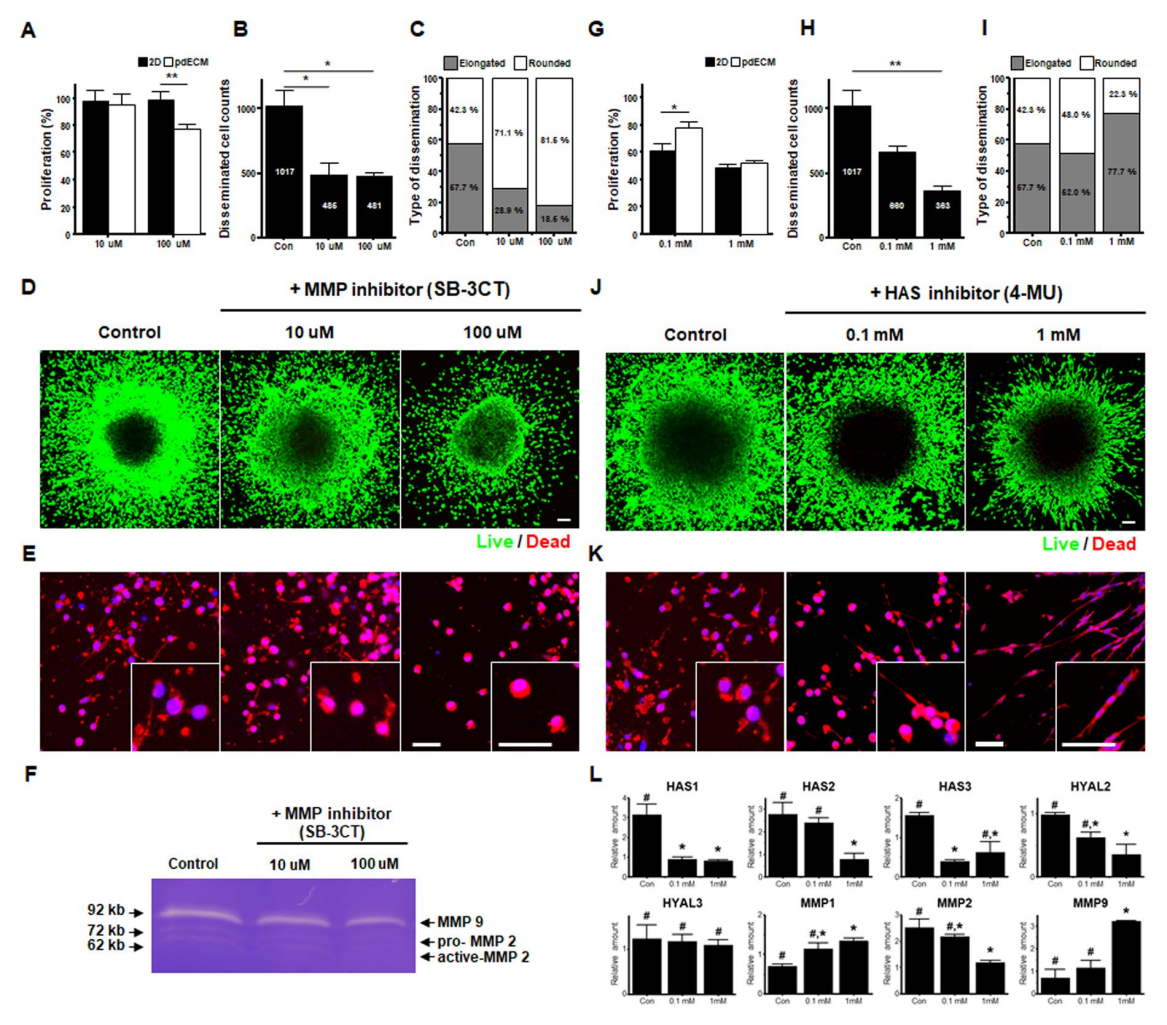
Influence of the ECM microenvironment on changes in response to various inhibitors in pdGCs. (**A**) The normalized viability of pdGCs in the presence of an inhibitor of matrix metalloproteinases 2 and 9 (MMP2/9; SB-3CT). (Asterisks indicate a significant difference statistically by student t-test, *p < 0.05; **p < 0.01). (**B**) Quantitative analysis of disseminated cells the in the presence of SB-3CT (n = 3~4; Asterisks indicate a significant difference statistically by ANOVA, *p < 0.05; **p < 0.01). (**C**) Quantitative analysis of the distribution by morphological type in the presence of SB-3CT. (**D**) Representative image of SB-3CT-treated pdGCs in pdECM hydrogels after 72 h invasion. (**E**) Magnified immunofluorescent images (F-actin) of invasion of pdGCs in the presence of SB-3CT treatment. (**F**) Effect of SB-3CT on pdGCs in pdECM hydrogels MMP-2 and -9 expression. Gelatin zymograms of pdGCs in presence of SB-3CT treatment. (**G**) Normalized viability of pdGCs in the presence of a hyaluronan synthase (HAS) inhibitor (4-MU) (Asterisks indicate a significant difference statistically by student t-test, *p < 0.05; **p < 0.01). (**H**) Quantitative analysis of disseminated cells in the presence of 4-MU (n = 3~4; Asterisks indicate a significant difference statistically by ANOVA, *p < 0.05; **p < 0.01). (**I**) Quantitative analysis of the distribution by morphological type in the presence of 4-MU. (**J**) Representative image of 4-MU treated pdGCs in pdECM hydrogels after 72 h invasion. (**K**) Magnified immunofluorescent images (F-actin) of invading pdGCs in the presence of 4-MU treatment (scale bar = 100 *μ*m). (**L**) Molecular expression profiles in presence of 4-MU. (n = 3~4; Different letter indicate a significant difference statistically by ANOVA, p < 0.05). The number of independent experiment (cell counting) were performed 6 times.

By PCR analysis, we confirmed that MMP9 and HAS2 were highly upregulated in pdGCs cultured within pdECM. In fact, both MMPs and HAS have been implicated as playing crucial roles in GBM invasiveness, thus causing ECM remodeling^19,20^. Therefore, we tested inhibitors of MMP2/9 and HAS to confirm the effect of those genes on cells with heterogeneous modes of invasion. The proliferation of pdGCs in the presence of an MMP inhibitor (SB-3CT) was relatively decreased in pdECM compared with a 2D environment at 100 µM, but not at 10 µM (Fig. 7A). Moreover, invasion of pdGCs was inhibited by SB-3CT (Fig. 7B, D). Interestingly, disseminated cells were significantly decreased in number and the morphology of invading cells was changed in pdECM. The distribution of morphological cell type dissemination was as follows: elongated, 57.7%; and rounded, 42.3% (without inhibition). In the presence of SB-3CT, the proportion of invading cells with a rounded morphology was increased in a dose-dependent manner (10 uM, 71.1%; 100 uM, 81.5%) (Fig. 7C, E). Furthermore, zymography demonstrated that the expression of MMP2 and MMP9 was slightly downregulated with higher doses of SB-3CT (Fig. 7F).

Next, we tested the effect of HAS inhibition on pdGCs invasion. 4-methylumbelliferone (4-MU) is an inhibitor of HA synthesis that blocks the production of HA components (and where glucuronic acid is a major component). We analyzed the proliferation rate and invasion of pdGCs after 4-MU treatment (0.1, 1 mM) for 72 h (Fig. 7G, J). pdECM displayed more resistance to 4-MU at 0.1 mM (proliferation rate: 78.45%), while there was no culture condition-dependent effect at a high concentration (1 mM). Interestingly, in the presence of 4-MU, the number of disseminated pdGCs decreased in pdECM, but most cells showed a morphological change from rounded to elongated (Fig. 7H, I, K). Magnified images of invaded pdGCs revealed elongated and thinner morphologies in a dose-dependent manner in pdECM (Fig. 7K). The molecular profiles of 4-MU-treated pdGCs were correlated with HAS inhibition (Fig. 7L), HAS expression was decreased in a dose-dependent manner. MMP2 and Hyal2 expression were significantly decreased, but there was no change in Hyal3 expression. Interestingly, MMP1 and MMP9 expression were significantly increased. These results suggest that inhibition of HAS promotes an alternative invasion strategy mediated by MMP.

On the other hands, such plasticity of cell invasion under enzymatic activity inhibition did not observe in the pure collagen matrix (Supplementary Figure 2). Although cells treated MMP inhibitor (0.1 mM) exhibited morphological change similar to the cells in pdECM, HAS inhibition did not provide the morphological change even at the high concentration. Therefore, we supposed that cells cultured in the collagen matrix is more sensitive and less resistant to these inhibitors than cells in pdECM.

## Discussion

Various methods have been used to study cell migration in GBM, such as 3D *in vitro* culture models, organotypic brain slice cultures, and *in vivo* mouse models have demonstrated different aspects of GBM invasion and migration^21,22^. In particular, 3D *in vitro* models have been developed extensively because such models offer a more precise and controlled environment, by modifying the properties of ECM. Although previously mentioned studies have provided insight into the phenotypic and invasive characteristics of GBM when cultured in 3D matrices, such as collagen I, Matrigel^23,24^, or chemically functionalized HA^13,21^, these matrices may not completely recapitulate the GBM-specific ECM microenvironment. Moreover, most *in vitro* studies, although demonstrating mesenchymal-type invasion in 3D models, typically fail to represent the heterogeneity and complexity of human tissue contexts sufficiently^18,25^.

Our decellularized pdECM-based 3D tumor model could recapitulate the specific ECM microenvironment of a GBM patient, including biochemical and biophysical characteristics. Using our model, we elucidated the impact of the ECM microenvironment on cell invasion. We found that pdGCs were more invasive and showed heterogeneous shapes, with elongated (58.7%) and rounded (41.3%) morphologies in pdECM hydrogels. In GBM, overexpressed HA and CD44 promote the invasive and infiltrative phenotype by activating a number of coordinated cellular programs^7,10,20,26^. From the characterization of pdECM, we identified that pdECM contains various ECM components such as HA and GAGs, which are the main component of the brain. Moreover, ECM-related genes including CD44 are unregulated in pdECM. These results show that HA-rich components in pdECM could influence invasion characteristics of pdGCs.

Interestingly, the different morphologies are accompanied by different migration trajectories and behaviors. In general, cancer cells that move through an ECM can be distinguished by invasion mode (two types). Mesenchymal mode relies on proteolytic degradation of the matrix; invading cells with a mesenchymal-like invasion show polarized extension of the leading edge of the cell in the direction of migration, as well as a spindle-shaped, elongated morphology^2^. Rounded cells show a more amoeboid mode of invasion, frequently is accompanied by cell blebbing and multiple or poorly defined protrusive cell edges. In the amoeboid mode, cells tend to migrate in the absence of proteolytic ECM degradation and to squeeze through the ECM space^2,27^. The morphology of invading cells accentuates direction of the protrusion as an important determinant of invading directionality. Provided that protrusion and adhesions formed by polarized cells are themselves directionally continuous, invading cells directionality will be continuous. Moreover, poorly defined protrusion or increased numbers of protrusion can bring on randomized migrati on^28–30^. Elongated pdGCs with long leading edge protrusions (uni-/bi-directional) showed directionally persistent migration. In contrast, rounded cells moved in a more random manner, having poorly defined protrusions or membrane blebbing-mediated movement.

It is well-known that ECM-related enzymes that degrade and synthesize the ECM are essential for tumor progression and in particular invasion^2,3,6^. According to our results, the ECM-related gene expression profiles of pdGCs cultured our 3D tumor models clearly depend on the ECM microenvironment (Fig. 6). Indeed, there is a close correlation between the enzymatic activities related ECM remodeling process and cell morphology (i.e., migration mode) during the invasion.

In addition to the co-existence of heterogeneous phenotypes of invasive pdGCs, we observed a morphological transition in each phenotype by blocking specific enzymatic activity. In our previous report, we demonstrated that ECM microenvironmental adaptation could allow a flexible invasion strategy for GBM cells cultured within a hyaluronic acid (HA)-rich 3D matrix. However, blockade of HAS-mediated invasion route was replaced by FAK-mediated invasion following degradation by MMPs^12^. Therefore, we hypothesize that mesenchymal- and amoeboid-like invasive strategies might display different enzymatic interactions with the surrounding matrix so that alterations influencing these interactions could lead to phenotypic transitions. In particular, the patient-derived ECM consists of HA, and the cells cultured in the pdECM could over-express HA-related genes (Hyal1/2, HAS2, CD44) and MMP9. Therefore, the role of enzymatic activities in cell invasion can be relevant for the heterogeneous invasion strategy in GBM cell invasion.

To confirm the effect of MMP- and HAS-mediated invasion on heterogeneous invasion characteristics, we tested SB-3CT (MMP2/9 inhibitor) and 4-MU (HAS inhibitor). Both inhibitors suppressed the proliferation and dissemination of GBM cells. For the MMP2/9 inhibitor, the disseminated cell type was mainly rounded, whereas, for the HAS inhibitor, the elongated morphology was predominant. This indicates that GBM cells can simply switch motility mode as an escape mechanism. GBM cell treated MMP2/9 inhibitor, used a rounded-amoeboid mode of invasion, due to detour the MMP-mediated invasion. On the other hand, inhibition of HA synthases (HAS) promoted the morphological transition from a rounded-amoeboid to elongated-mesenchymal morphology. In addition to this morphological change, in the quantitative PCR (qPCR) analysis, HAS family expression was significantly reduced but the expression levels of MMP1 and MMP9 were increased. According to our results, it appears that MMP-mediated proteolysis of the cell cultured in pdECM plays a major role in GBM cell invasion by the elongated-mesenchymal phenotype, while HAS-mediated cell migration facilitates rounded-amoeboid phenotype in GBM cell invasion. Presumably, newly synthesized HA can induce local hydration of ECM and create spaces through which GBM cells may migrate and invade in an amoeboid manner. Therefore, our results indicated that GBM cells alter their invasion route to achieve aggressive motility in a 3D microenvironment. Consequently, because GBM cells rapidly change their migratory mode according to the specific environment, therapeutic blockage of one mechanistic pathway may trigger alternative mechanisms of invasion and thus lead to resistance.

Our approach to recapitulate physiologically relevant ECM microenvironment in tumor model utilizes a conventional decellularization method to obtain organ-specific ECM components. Other methods of construction of *in vitro* brain tumor model have been reported, including HA-collagen interpenetrating network (IPN) hydrogel^12^, HA incorporated gelatin and poly(ethylene glycol) (PEG) based hydrogel^13^, and methacrylated-HA hydrogel^21^. Unlike these matrices, using decellularized patient-derived ECM provides a more relevant microenvironment with major components of brain tumor ECM (HA, GAG, and other ECM proteins), as well as the microarchitecture. Our results show the effects of patient-derived ECM to facilitate the invasion and dissemination and also demonstrate the alteration of invasion strategy by blocking major enzymatic-activity for ECM remodeling. Thus we believe that organ-specific ECM-based 3D tumor models could provide a better understanding of cancer cell-ECM interactions and inform the choice of ECM-targeted therapeutic options.

## Materials and Methods

### Preparation of pdECM & pdECM Hydrogel Formation

All patients provided written informed consent, and permission for specimen sampling and evaluation was obtained from the Institutional Review Boards at our institutes (Yonsei University Health System, Severance Hospital, Institutional Review Board (4-2014-0649), The Collaborative Institutional Training Initiative (CITI) Program at the University of Miami (K-2014-13752477)), as specified in the Declaration of Helsinki. Human brain tissues were freshly obtained from the operation room (Severance Hospital, Yonsei University College of Medicine) during the transcortical approach for surgery. Patient tissues were cut into small pieces (3 mm × 3 mm × 3 mm). Subsequently, to remove the cellular component from patient derived brain tissues decellularizing solution (0.1% (v/v) ammonium hydroxide (Sigma-Aldrich, St. Louis, MO, USA) and 1% (v/v) Triton X-100 (Sigma-Aldrich) in distilled water) was treated for 2 days. After that decellularized brain tissue-derived brain ECM (pdECM) was washed using distilled water to remove the detergent solution and cellular residue. Finally, pdECM was lyophilized and stored at −20 °C until use. For the subsequent experiment, lyophilized pdECM was ground and then enzymatically digested with pepsin (1 mg/ml; Sigma-Aldrich) in HCl (0.01 N; Sigma-Aldrich) for 2 days at room temperature until visible ECM particle disappeared. The final concentration of the pdECM solution was 20 mg/ml. To make hydrogels, the pdECM solution was mixed with 10× PBS, dilute to the desired final concentration (20 mg/ml) with ice-cold distilled water and adjust to neutralize pH (7.0) by NaOH (1 M; Sigma-Aldrich). After that, the pdECM solution was blended with collagen solution (4 mg/ml; BD biosciences, Franklin Lakes, NJ, USA) 10:1 (v/v). Finally, the pre-gel solution was incubated for 1 hr at 37 °C.

### Characterization of pdECM

For histological analysis, native tissue and pdECM were embedded in Paraplast (Leica Biosystems, St Louis LLC, Netherlands) and then the sample was sectioned into 5 µm. To check the removal of cellular components and general structure of tissues, sectioned samples were stained with hematoxylin and eosin (H&E). Permanently, mounted slides were observed and photographed using a microscope equipped with a digital imaging system (DP72, Olympus, Tokyo, Japan). The quantification of DNA content in pdECM was determined by DNA extraction kit (Bioneer, Daejeon, Korea) and measuring the DNA concentration by ND-1000 Spectrophotometer (Thermo Scientific, Wilmington, USA). To determine the content of GAGs in pdECM and native tissue, 1,9-dimethyl methylene blue (DMMB) (Sigma-Aldrich) assay was used as previously described^31^. The HA content in the pdECM and native tissue was quantified by carbazole assay as previously described^32^. The collagen content in the pdECM and native tissue was quantified by hydroxyproline assay as previously described^33^. The Bohlin Advanced Rheometer (Malvern Instruments, Worcestershire, U.K.) was used for characterization of the mechanical properties of pdECM hydrogels. The rheological features of pdECM hydrogels were measured in a frequency sweep mode. The storage modulus (G’) and loss modulus (G”) of the pdECM hydrogel were recorded at a given 1% strain. The elastic modulus of pdECM hydrogels was calculated at 1 Hz.

### Cell culture

Tumorspheres (TS) were directly established from fresh GBM tissues as approved by the institutional review board of Severance Hospital, Yonsei University College of Medicine (4-2012-0212). For isolation of TS from GBM specimens, we followed previously published methods for TS isolation from human brain^17,34^. The patient-derived GBM cells (pdGCs; TS) were isolated and cultured in 2D, collagen hydrogel, and pdECM environments, respectively. For culture, pdGCs (TS13-20) were cultured in complete TS media composed of DMEM/F-12 containing B27 supplements (1×; Invitrogen, San Diego, CA, USA), 20 ng/ml of basic fibroblast growth factor (bFGF; Sigma-Aldrich), 20 ng/ml of epidermal growth factor (EGF; Sigma-Aldrich), and 50 U/ml penicillin, 50 mg/ml streptomycin.

### Invasion assay

The polydimethylsiloxane (PDMS)-based microwells (diameter and depth of microwells: 6 mm and 500 μm) was utilized for invasion assay. PDMS microwells were treated with poly (ethyleneimine) (1%; Sigma-Aldrich) solution for 10 minutes followed by glutaraldehyde (0.1%; Sigma-Aldrich) for 30 minutes and then washed overnight with PBS (Invitrogen) to create a matrix-adherent surface. For evaluating the invasive ability, the pdGCs were embedded within pdECM hydrogels (10:1 ratio (v/v) with collagen) for 72 hours. The dynamic invasion of pdGCs was monitored using an inverted confocal laser-scanning microscope (Ti-E; Nikon, Tokyo, Japan), by whole depth of 3D platform. The live images of pdGCs in the 3D matrix were obtained using the confocal microscope (Nikon). Subsequently, the live images were analyzed using NIH ImageJ imaging software (NIH ImageJ; National Institutes of Health, Bethesda, MD). Each experiment repeated over the 10 times.

### Immunocytochemistry

pdGCs that cultured in collagen and pdECM environments for 72 hours were fixed in 4% paraformaldehyde (PFA). After fixation with PFA, pdGCs were permeabilized with Triton X-100 (0.3%.; Sigma-Aldrich) F-actin was stained with rhodamine-conjugated phalloidin (Sigma-Aldrich) for 1 hr and nucleus was stained with DAPI (Sigma-Aldrich) for 5 minutes. And then the samples were washed with PBS and mounted with Prolong® Gold Antifade Reagent (Molecular Probes, Oregon, USA). Samples were observed using a confocal microscopy equipped with a digital imaging system (Nikon).

### Quantitative Real-Time Polymerase Chain Reaction (qRT-PCR)

After 3 days, the gene expression of fully-invaded TSs within biomimetic environment was examined with qRT-PCR. Total RNA was extracted from each sample (at least n = 6 per each group) according to conventional RNA extraction protocol. Briefly, the samples were prepared with RNA isolation reagent and extracted with chloroform after 10 min, 14,000 rpm centrifugation. Reverse transcription was carried out using the cDNA Synthesis kit (Bio-rad, Richmond, CA, USA). qRT-PCR was performed using a PCR System (Bio-rad) with PCR Master Mix (Toyobo, Osaka, Japan). Gene expression of each marker was quantified using signal amplification of SYBR green (Bio-rad) for CD44, hyaluronidase (HYAL) 1, 2, 3, hyaluronan synthase (HAS) 1, 2, 3, matrix metalloproteinase (MMP) 1, 2, 9 and glyceraldehyde-3-phosphate dehydrogenase (GAPDH). The level of gene expression was determined with the comparative Ct method in which the target genes were normalized to the endogeneous reference (GAPDH). The forward/reverse sequences of primers used for experiments was listed in Table 1.

**Table 1:** Primer sequences used in qPCR analysis.

Gene	Forward primer (5′ → 3′)	Reverse primer (3′ → 5′)
GAPDH	GTATGACAACAGCCTTCAAGAT	AGTCCTTCCACGATACCAAA
CD44	CTCTCTCCCTCCACTTCAC	GCCTAATGTCCAGTTTCTTTCA
HAS1	GGTGGGGACGTGCGGATC	ATGCAGGATACACAGTGGAAGTAG
HAS2	GTGGATTATGTACAGGTTTGTGA	TCCAACCATGGGATCTTCTT
HAS3	CTCTACTCCCTCCTCTATATGTC	AACTGCCACCCAGATGGA
HYAL1	CATCAAGGAGTATATGGACAC T	CAGGGTTAAGGAGGAGGA
HYAL2	GATTACCTGACACGGCTG	GAAACTGTTGGTGCTGAGA
HYAL3	TTTCCCTGCTGCCACTTT	CTTGGGAGGGTTGACTGTAA
MMP1	AGCTAGCTCAGGATGACATTGATG	GCCGATGGGCTGGACAG
MMP2	GACGGTAAGGACGGACTC	ACTTCACACGGACCACTT
MMP9	TGGGCTACGTGACCTATGACAT	GCCCAGCCCACCTCCACTCCTC

### Cell viability assay

Effects of drug (4-MU, SB-3CT) on the survival of pdGCs were determined by WST-1 method (Roche Diagnostics, Mannheim, Germany). Briefly, pdGCs cultured in invasion assay platform were incubated with the WST-1 solution. After incubated samples at 37 °C for 3 h, the absorbance (O.D.) was measured at 450 nm using a microplate reader (Bio-rad). The viability was measured using the absorbance, compared to control group.

### MMP zymography

The SB-3CT treated pdGCs media was mixed with sample buffer and loaded without boiling to a 10% polyacrylamide gel (Bio-rad) containing gelatin (1 mg/ml; BD biosciences) and 0.1% SDS (Sigma-Aldrich). After electrophoresis, the gels were incubated in renaturing buffer (Thermo Fisher, Waltham, MA, USA) at room temperature for 0.5 hr and then incubated in developing buffer (Thermo Fisher) at room temperature for 16hr. MMP activity was visualized by staining with Coomassie Brilliant Blue R-250 (Bio-rad).

### Statistical analysis

Data are presented as the mean with standard deviations (SD). Level of significance for comparisons between each sample was calculated by using one-way analysis of variance, followed by Student’s t-test. A significant difference in statistics was considered at p < 0.05.

## Electronic supplementary material

Supplementary information

Supplementary Movie 1a

Supplementary Movie 1b

Supplementary Movie 1c

Supplementary Movie 1d

Supplementary Movie 1e

Supplementary Movie 2a

Supplementary Movie 2b

Supplementary Movie 2c

Supplementary Movie 2d

Supplementary Movie 2e

Supplementary Movie 2f
