# Supplementary material for: The mode and dynamics of glioblastoma cell invasion into a decellularized tissue-derived extracellular matrix-based three-dimensional tumor model
Source: Sci Rep. 2018 Mar 15;8:4608. doi: 10.1038/s41598-018-22681-3 (PMC5854588; doi:10.1038/s41598-018-22681-3)
Supplement: Supplementary file 1 — Supplementary information [file 41598_2018_22681_MOESM1_ESM.pdf]

Supplementary Information

The mode and dynamics of glioblastoma cell invasion into a decellularized tissue-derived extracellular matrix-based three-dimensional tumor model

IlKyoo Koh[a], Junghwa Cha[a], Junseong Park[b], Junjeong Choi[c], Seok-Gu Kang[b,], Pilnam Kim[a, *]*

[a]*Department of Bio and Brain Engineering, KAIST, Daejeon 34141, Korea*

[b]*Department of Neurosurgery, Brain Tumor Center, Severance Hospital, Yonsei University College of Medicine, Seoul 03722, Korea*

[c]*Department of Pharmacy, College of Pharmacy, Yonsei Institute of Pharmaceutical Sciences, Yonsei University, Incheon, Republic of Korea*

*Correspondence: Pilnam Kim, Ph.D.

e-mail: pkim@kaist.ac.kr

Seok-Gu Kang, MD, Ph.D

e-mail: seokgu9@gmail.com

Supplementary Figures

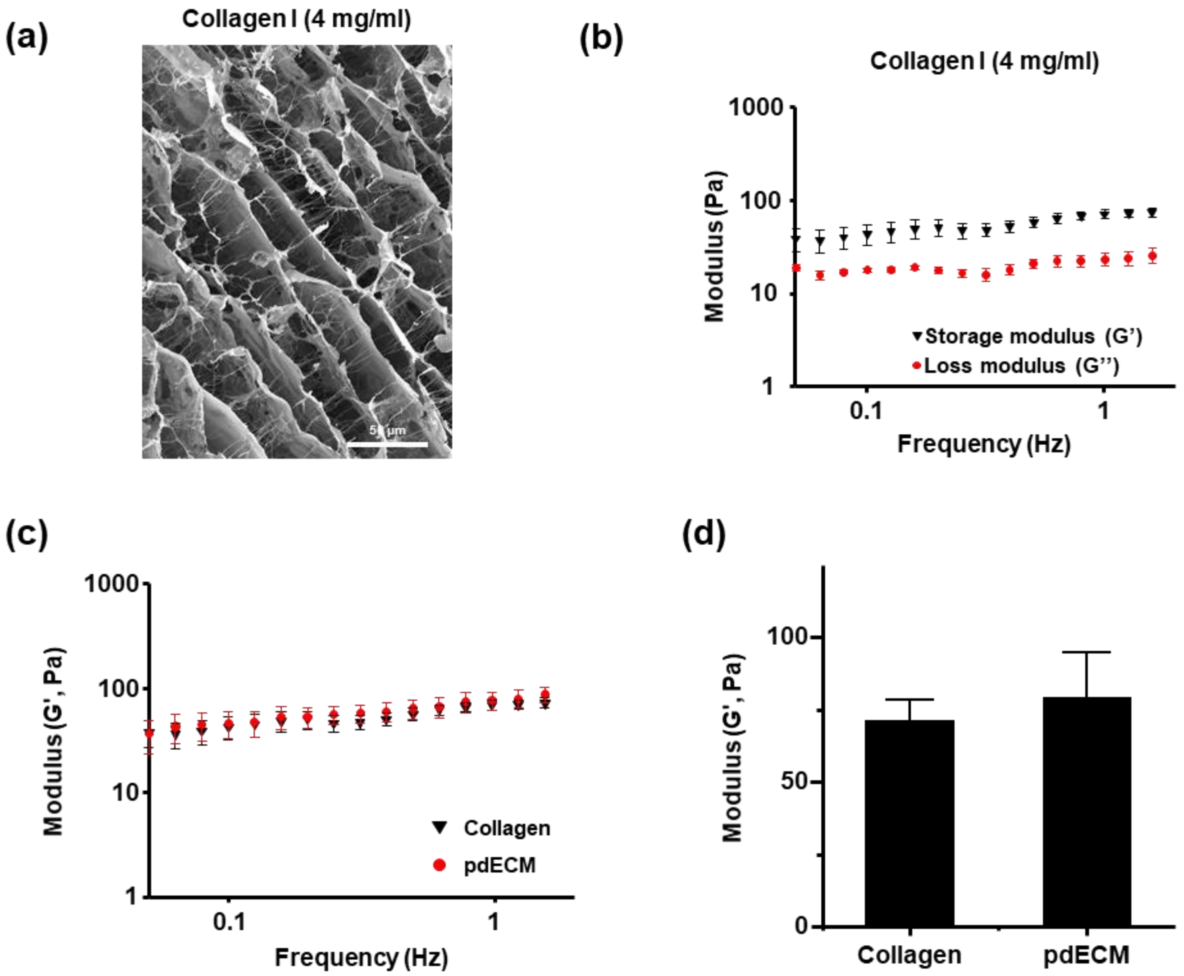

Supplementary Figure 1. (a) SEM image of collagen hydrogel. (b) Mechanical property of collagen hydrogel. (c) Elastic modulus of collagen and pdECM hydrogel (d) Average elastic modulus of collagen and pdECM hydrogels at a frequency of 1 Hz.

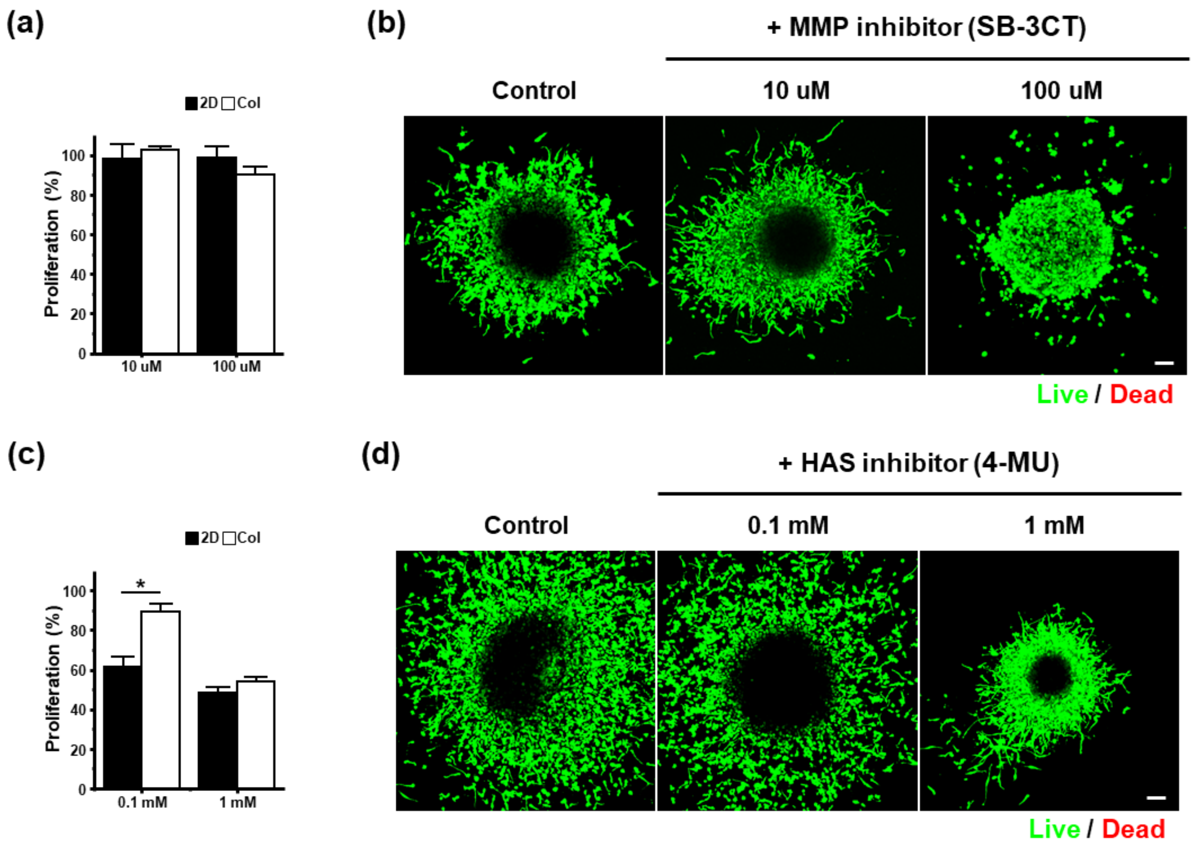

Supplementary Figure 2. (a) The normalized viability of pdGCs in the presence of an inhibitor of matrix metalloproteinases 2 and 9 (MMP2/9; SB-3CT). (b) Representative image of SB-3CT-treated pdGCs in collagen hydrogels after 72 h invasion. (c) Normalized viability of pdGCs in the presence of a hyaluronan synthase (HAS) inhibitor (4-MU) (*, $p<0.05$; **, $p<0.01$). (d)) Representative image of 4-MU treated pdGCs in collagen hydrogels after 72 h invasion. (n = 3~4; Asterisks indicate a significant difference statistically by student's t-test, *$p < 0.05$).
